# Remote intervention using smartphone for rural women suffering from premenstrual syndrome: A propensity score matched analysis

**DOI:** 10.1097/MD.0000000000011629

**Published:** 2018-07-20

**Authors:** Ning Chai, Ying Wu, Miao Zhang, Wen-Bin Wu, Hui Zhang, Feng-Wei Kong, Ying Zhang

**Affiliations:** a Department of Nephrology and Critical Care Medicine; b Department of Thoracic Surgery, Xuzhou Central Hospital Affiliated to Southeast University; c Department of General Surgery, Xuzhou Infectious Disease Hospital, Xuzhou, China.

**Keywords:** internet-based intervention, premenstrual syndrome (PMS), remote intervention, rural women

## Abstract

Timely treatment of premenstrual syndrome (PMS) is not always available for rural women, because the local medical resources are insufficient. The efficacy of remote intervention by smartphone on PMS has not been confirmed.

A retrospective analysis was performed on rural PMS patients between January 2014 and December 2015. After a propensity score matched analysis, 60 patients were enrolled and evenly divided into remote group and outpatient group. Multidisciplinary therapy including cognitive-behavioral therapy (CBT), oral medication, and physical exercise education was used individually, in accordance with their symptoms evaluated by Daily Record of Severity of Problems (DRSP) questionnaire. Patients in remote group utilized WeChat software by smartphone for therapy guidance, while those in outpatient group attended face-to-face interview. Their DRSP scores in 5 new menstrual cycles after therapy were recorded. Then, they were followed up for 1 year.

Total DRSP scores of the cases in both groups after initial intervention were less than those before therapy (*P* < .001), without group difference (*P* > .05). However, patients in remote group indicated a higher satisfactory rate than the outpatient group (*P* = .03). On the 1-year follow up, patients in both groups demonstrated similar DRSP scores (*P* = .07), but the satisfactory rate in remote group was encouragingly higher than that in the outpatient group (*P* = .02).

The efficacy of remote intervention using smartphone on PMS is noninferior to traditional outpatient visits. Nevertheless, high-quality trials are needed.

## 1 Introduction

Premenstrual syndrome (PMS) is characterized by recurrent affective symptoms during the luteal phase due to unknown etiology.^[1]^ It is reported that more than 90% of women are affected by one or more symptoms of PMS.^[2]^ Premenstrual dysphoric disorder (PMDD) is a severe form of PMS, and nearly 1.3% of the women meet the criteria of PMDD.^[3]^ The therapeutic methods for PMS include serotonergic antidepressants, combined oral contraceptives, physical exercise, lifestyle modification, cognitive-behavioral therapy (CBT), supplements, and herbal medicine.

However, a cross-sectional study shows that only 48.0% participants with PMS seek medical therapy.^[4]^ Furthermore, the pain and suffering of women have been dismissed, minimized, and negated historically.^[5]^ The local medical resource is sometimes unavailable for rural patients. Thanks to the popularization of smartphone and internet, patients in rural areas could get free access to the online medical service. Nevertheless, the efficacy of remote intervention on PMS has not been clarified yet. Herein, a retrospective study is presented to evaluate the efficacy of intervention using smartphone for PMS patients.

## 2 Patients and methods

A retrospective analysis was performed on the PMS patients who asked for therapy at outpatient clinic or by smartphone between January 2014 and December 2015. Written informed consent was obtained from each participant before this study, and it was approved by the Institutional Review Board of Xuzhou Central Hospital. All participants were given an explanation of their rights and privacy protection.

### 2.1 Inclusion and exclusion criteria for data collection

The diagnosis of PMS is based on the self-report Daily Record of Severity of Problems (DRSP).^[6]^ Each item of DRSP is scored from 1 to 6 points, with 6 indicating the most severe problems.^[6]^ A day is considered symptomatic if the patient had at least 3 symptoms, each with a score of at least 3.

**Figure 1:**
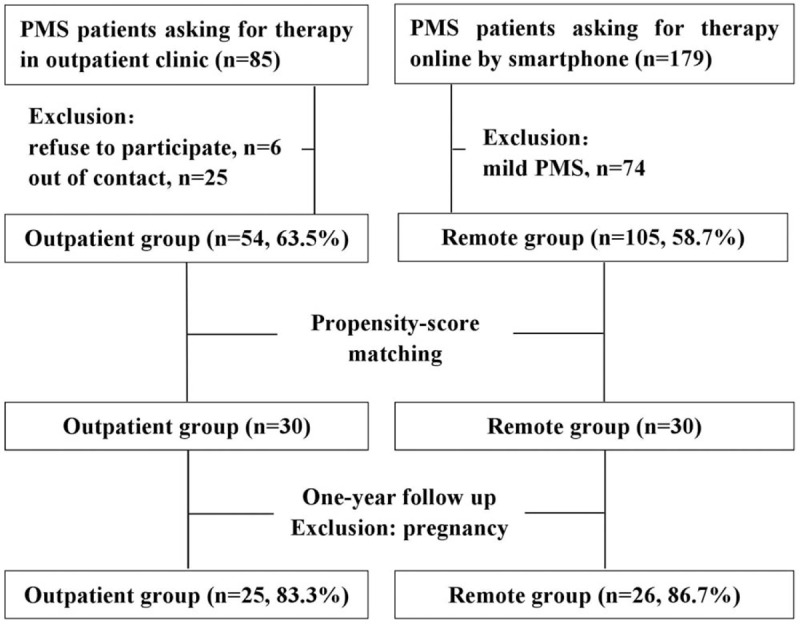
The flowchart of this retrospective study.

The cases in this study were staged according to the reported diagnostic criteria of PMS for Chinese patients.^[7]^ The PMS patients with regular menstrual cycles of 25 to 32 days without physical or mental abuse or therapy during the past 3 months were included.^[8]^ Patients with irregular menstrual cycle, menstrual abnormalities, pregnancy, lactating, tumors, breast or other organic diseases, taking psychotropic or contraceptive pills 3 months before the study, or a history of chronic somatic pain were excluded.^[7,9]^ Patients who refused to participate or out of contact were also excluded. The flow chart is indicated as Fig. 1.

The following variables of the patients were evaluated: age, gender, body mass index (BMI), smoking index, menarche age, length of menstrual cycle, days of bleeding menses, educational level in school, marital status, working status, and family income per year. The patients who visited outpatient clinic for help showed moderate-to-severe symptoms, including breast tenderness, headache, and long-time fatigue. In contrast, many cases who asked for medical assistance by smartphone demonstrated mild symptoms. Therefore, a propensity score matched analysis was utilized. The values for matching included severity of PMS on admission, preexisting education level in school, working status, family income level, and marriage status of these patients. At last, 60 PMS participants aged 18 to 30 years were enrolled, with 30 patients in each group.

### 2.2 Intervention procedures

The cases in outpatient group received therapy guidance in face-to-face interview, while those in remote group received advices using smartphone app of WeChat. Multidisciplinary regimen was administrated individually in both groups. It mainly includes CBT, oral medication of sertraline hydrochloride, citalopram or escitalopram, and physical exercise of Baduanjin, Tai Chi, or Yoga. All these patients received demonstrative videos of Yoga, Tai Chi, or Baduanjin. They mastered one of them and practiced daily.

In this study, all the patients received first-line therapy as reported,^[6]^ using symptom-triggered and self-guided procedure. Patients with moderate PMS symptoms were treated by CBT combined with physical exercise, while the oral pills were added for severe PMS patients with DRSP score of 56 to 70. The starting dose of sertraline hydrochloride was 50 mg once per day, during the symptomatic days in 5 menstrual cycles.

All the participants were informed of the meaning of each item of the DRSP form and how to fill in the form daily according to their symptoms during the luteal phase. Their guardians or caregivers are necessary for assistance to complete the DRSP questionnaire, when the patients do not want to finish the questionnaire on time. Clinical outcomes were assessed by both self-report questionnaires and interviews to decrease bias.

### 2.3 Outcome measures and follow-up

The total DRSP scores during the 5 premenstrual days were calculated. The primary outcome was the total DRSP scores of the patients after therapy, and the secondary outcome was their satisfaction rate. The patients in both groups were followed up for 1 year after the initial treatment, using normal telephone or WeChat. The DRSP scores and satisfactory rate of the cases in each group were re-evaluated subsequently. The cases who were pregnant during the follow-up were excluded.

### 2.4 Statistical analysis

Continuous variables were recorded as means ± SD (xx00AF;x00B1;s). Student *t* test, analysis of variance (ANOVA), or Mann–Whitney *U* test was performed for comparison of quantitative data, while Yates-corrected Chi-square or Fisher exact test was used for categorical variables or enumeration data. SPSS, version 19.0 (IBM SPSS, Armonk, NY) was used in this study. *P* < .05 was considered statistically significant.

## 3 Results

The baseline characteristics, including age, BMI, menarche age, length of menstrual cycle and bleeding menses, smoking history, educational level, marital status, working status, and DRSP scores on admission of the 2 groups were comparable after propensity-score matching, as summarized in Table 1.

**Table 1:**
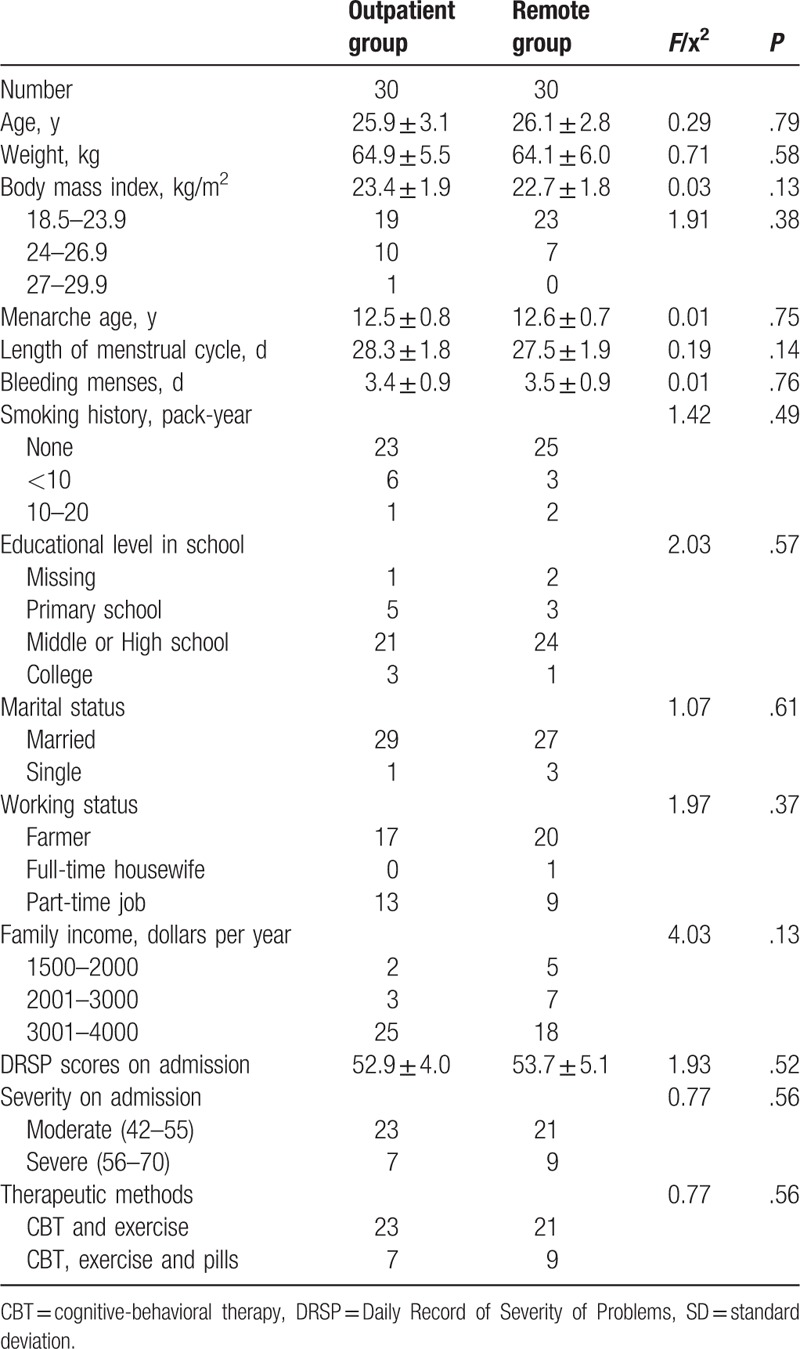
Baseline characteristics of the patients before therapy.

**Figure 2:**
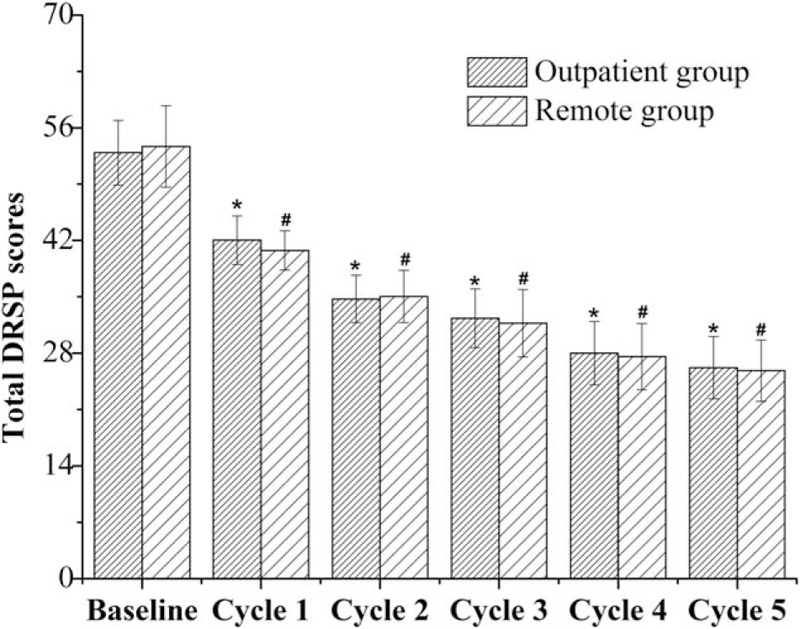
Total DRSP scores of the patients before and after treatment. ^∗^*P* < .001, as compared with the baseline of outpatient group; ^†^*P* < .001, as compared with the baseline of remote group.

The DRSP scores of the cases in each group decreased over time during the 5 premenstrual days after treatment (*P* < .001), as compared with the baseline. However, group difference was not indicated from cycle 1 to cycle 5 after therapy (*P* = .13, .44, .38, .89, .63, respectively), as shown in Fig. 2. In detail, 27 participants in remote group and 25 cases in outpatient group responded to the therapy on the first cycle after treatment. Besides, PMS remission was attained by 24 patients in remote group and 23 cases in the outpatient group on the fifth cycle. Moreover, the patients in remote group showed higher satisfaction rate (90.0% vs 66.7%, *P* = .03) than the counterparts.

**Figure 3:**
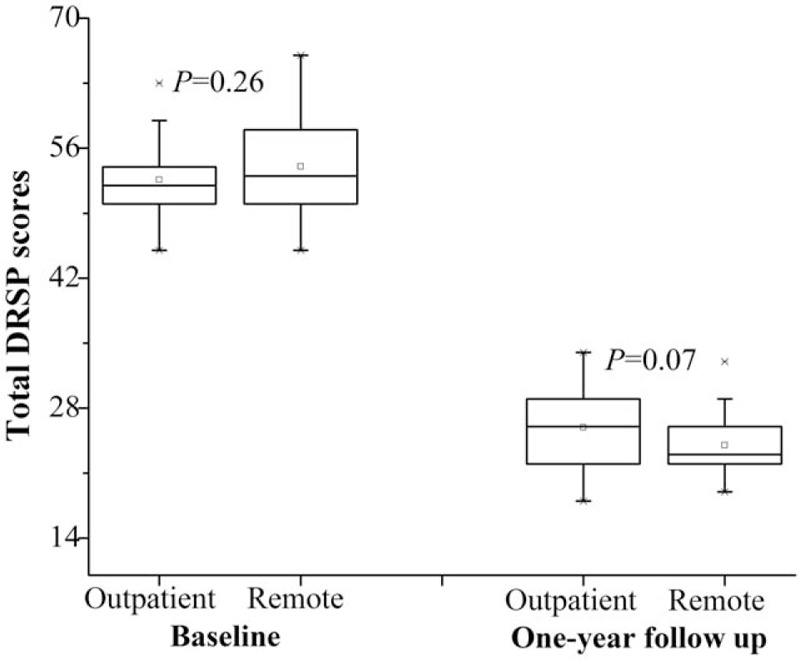
DRSP scores of the patients on 1-year follow-up.

On the 1-year follow-up after initial treatment, 5 participants in the outpatient group and 4 cases in the remote group withdraw for pregnancy. Finally, 25 patients in the outpatient group and 26 cases in the remote group fulfilled the interview by telephone or WeChat. Patients in both groups indicated comparable compliance and persistent efficacy without group difference (*P* = .07), as shown in Fig. 3. However, the patients in remote group demonstrated higher satisfaction rate (88.5% vs 56.0%, *P* = .02) than those in outpatient group.

## 4 Discussion

A large percentage of the rural PMS patients were interviewed during the therapy. The major reasons why they did not attend hospital for therapy before were limited family income and unawareness of their health status. Although the admission rate of rural patients is increased after the medical reform, their psychosocial burden is still high because of their insufficient family income; however, there is no exact statistic about this issue at present. Besides, their unawareness of self-protection was probably ascribed to their limited education, including but not limited to the fundamental knowledge of physiology and medicine. Furthermore, many patients did not ask for therapy because their physical examination and laboratory test were normal. Thus, the influence of PMS on their quality of life was somewhat underestimated.

The efficacy of internet-based, remote intervention for PMS is tentatively indicated in this study. Several issues need to be elucidated by further high-quality studies.

First, PMS encompasses a vast array of physical and psychological symptoms such as depression, anxiety, irritability, loss of confidence, and mood swings during the luteal phase of the menstrual cycle.^[10]^ The onset of PMS is correlated with more consumption of egg yolk, greater alcohol intake, poorer sleep quality, higher likelihood of psychiatric morbidity, family history of dyslipidemia, and a higher serum cholesterol level.^[11]^ Besides, violence victimization is prevalent among PMS women; meanwhile, childhood emotional and physical abuse increase the risk of PMS.^[12]^

Patients with severe PMS may benefit from multidisciplinary, integrated holistic approach. The lowering of serotonin can give rise to PMS-like symptoms, and agents that augment serotonin are efficacious.^[13]^ Serotonergic antidepressants selective serotonin reuptake inhibitors (SSRIs) are highly effective as first-line pharmacologic therapy for PMS.^[14]^ Abrupt treatment cessation is not associated with discontinuation symptoms.^[15]^ The psychiatric medications venlafaxine, duloxetine, alprazolam, and buspirone are also effective for PMS. However, the use of these treatments remains limited due to their potential side effects and expense.^[16]^ Preferred prescription treatment of PMS in UK has changed from progestogen to SSRIs and combined oral contraceptives.^[17]^ It is reported that Baduanjin exercise could improve the mental and physical symptoms of PMS.^[7]^ The efficacy of CBT or serotonergic antidepressants alone is not satisfactory on PMS; thus, future research should possibly focus on combined approaches.^[18]^

Many women experience difficulty anticipating the onset of symptoms, or attribute symptoms to environmental stressors rather than premenstrual disorders.^[15]^ A high percentage of PMS women are dissatisfied with their actual treatment.^[9]^

Second, the evidence regarding the efficacy of psychological therapies delivered by internet is insufficient.^[19]^ The internet-based self-help program for PMS is not available. Psychological interventions such as CBT may have beneficial effects in managing PMS symptoms.^[20,21]^ This study shows that remote intervention is as effective as outpatient visit for PMS patients, followed by better compliance and higher satisfactory rate. Thus, the therapeutic information could be delivered by internet instead of outpatient visit. Furthermore, it is reported that people in rural areas have poorer health outcomes than their urban counterparts,^[22]^ while urban women report more severe psychological symptoms than rural women.^[23]^ Education regarding menstrual-related matters would have a positive impact on nurturing a positive symptom expression and treatment-seeking attitude.^[24]^ In addition, spouse's educational intervention about supportive behaviors can reduce PMS symptoms.^[25]^ Couple-based CBT interventions may have a greater positive impact on behavioral coping for PMS women, as compared with one-to-one modality.^[26]^

On the basis of our primary findings, the past educational level of the rural PMS patients is probably positively correlated with their compliance to treatment. Actually, 29.4% (25/85) of the rural PMS patients in outpatient clinic group were lost to follow-up, while the cases in smartphone group demonstrated a notably better compliance with the treatment. Therefore, the implementation of education program regarding the diagnosis and therapy of PMS into daily life may be helpful. It could be delivered by television, radio, or digital versatile disc. The self-help DRSP questionnaire could be illustrated as pictures to be more applicable. Meanwhile, health care reform should pay attention to reducing the financial burden of rural patients, which is a major factor in therapeutic decision-making.

### 4.1 Limitations of this study

This study is limited by its small sample size after propensity-score matching, bias, and under-reporting due to remote evaluation. Only patients with moderate-to-severe PMS were included for analysis. The patients could communicate with their doctors or nurses through the free WeChat just as face-to-face interview. But the fidelity of online procedure to CBT has not been elucidated yet. Their sexual life quality and family relationship may be significantly correlated with the severity of PMS, but they were not evaluated in this study for privacy issue. Furthermore, although the knowledge about PMS probably influence their attitude toward therapy, the exact effect of education on their compliance to therapy is unclear. Moreover, this study did not compare the symptom scores for depression, anxiety, decreased interest, and less participation in social activities, respectively. In addition, the role of CBT and physical exercise was not demonstrated independently. A reviewer advised that this pilot study could be improved with larger population, with the aim to clarify the actual effect of sexual life quality, education, nurture, family income, and CBT on their response to therapy.

## 5 Conclusion

The efficacy of remote intervention by smartphone on PMS is noninferior to face-to-face outpatient visit. This procedure could serve as an alternative of hospital admission for rural PMS patients. High-quality studies with long-term follow-up are needed to verify our findings.

## Author contributions

**Conceptualization:** Ning Chai.

**Data curation:** Miao Zhang, Wen-Bin Wu, Feng-Wei Kong.

**Formal analysis:** Ying Wu.

**Funding acquisition:** Hui Zhang.

**Methodology:** Ying Zhang.

**Project administration:** Hui Zhang.

**Software:** Ying Wu, Wen-Bin Wu.

**Writing – original draft:** Ning Chai, Ying Wu, Miao Zhang, Ying Zhang.

**Writing – review & editing:** Ning Chai, Miao Zhang, Wen-Bin Wu, Feng-Wei Kong, Ying Zhang.
